# High Frequency of CD4^+^CXCR5^+^ TFH Cells in Patients with Immune-Active Chronic Hepatitis B

**DOI:** 10.1371/journal.pone.0021698

**Published:** 2011-07-07

**Authors:** Junyan Feng, Lu Lu, Cong Hua, Ling Qin, Pingwei Zhao, Juan Wang, Ye Wang, Wanyu Li, Xiaodong Shi, Yanfang Jiang

**Affiliations:** Department of Central Laboratory, the Second Part of First Hospital, Jilin University, Changchun, China; University of Cape Town, South Africa

## Abstract

**Background:**

T follicular helper (TFH) cells are a special subpopulation of T helper cells and can regulate humoral immune responses. This study examined whether the frequency of CD4^+^CXCR5^+^ TFH cells could be associated with active immunity in chronic hepatitis B (CHB) patients.

**Methodology and Findings:**

The frequencies of peripheral blood CD4^+^CXCR5^+^ TFH cells, inducible T cell costimulator (ICOS), and/or programmed death 1 (PD-1) positive CD4^+^CXCR5^+^ TFH cells in immune-active (IA), immune-tolerant (IT) CHB, and healthy controls (HC) were characterized by flow cytometry analysis. The effect of adevofir dipivoxil treatment on the frequency of CD4^+^CXCR5^+^ TFH cells, the concentrations of serum IL-2, IFN-γ, TNF-α, IL-4, IL-6, IL-10, IL-21, ALT, AST, HBsAg, HBsAb, HBeAg, HBeAb and HBV loads in IA patients were determined. The potential association of the frequency of CD4^+^CXCR5^+^ TFH cells with clinical measures was analyzed. In addition, the frequency of splenic and liver CD4^+^CXCR5^+^ TFH cells in HBV-transgenic mice was examined. We found that the frequency of CD4^+^CXCR5^+^ TFH cells in IA patients was significantly higher than that of IT patients and HC, and the percentages of CD4^+^CXCR5^+^ TFH in IA patients were positively correlated with AST. Furthermore, the percentages of ICOS^+^, PD-1^+^, and ICOS^+^PD-1^+^ in CD4^+^CXCR5^+^ TFH cells in CHB patients were significantly higher than that of HC. Treatment with adefovir dipivoxil reduced the frequency of CD4^+^CXCR5^+^ TFH, PD-1^+^CD4^+^CXCR5^+^ TFH cells and the concentrations of HBsAg and HBeAg, but increased the concentrations of HBsAb, HBeAb, IL-2 and IFN-γ in IA patients. Moreover, the frequency of splenic and liver CD4^+^CXCR5^+^ TFH cells in HBV-transgenic mice was higher than that of wild-type controls.

**Conclusions:**

These data indicate that CD4^+^CXCR5^+^ TFH cells may participate in the HBV-related immune responses and that high frequency of CD4^+^CXCR5^+^ TFH cells may be a biomarker for the evaluation of active immune stage of CHB patients.

## Introduction

HBV infection is a global health concern and an economical burden, affecting approximately 400 million people worldwide [1]. Many patients infected with HBV progress into chronic hepatitis B (CHB) and can develop end-stage consequences (cirrhosis and hepatocellular carcinoma) [1]. There are about 130 million patients with HBV infection and 20% of them develop CHB in China [1]. During the HBV-infection, the interaction between replicating noncytopathic virus and dysregulatory host antiviral immunity determines the outcome [2]. Furthermore, the responses of individual patients to anti-virus drug treatment are also variable. Apparently, host and viral factors contribute to variable biochemical, virological, and histological profiles at different stages of the process of CHB [2]–[5]. Previous studies indicate that dynamic interactions between the virus, hepatocytes, and the host immune system may determine viral persistence and disease progression, which are displayed in distinct successive phases [4]. Individuals with HBV infection at immune-tolerant (IT) phase can display effective replication of HBV, but with no obvious liver damage and normal levels of serum alanine aminotransferase (ALT). However, others at immune-active phase (IA) can exhibit severe liver damage with abnormal levels of ALT, but reduced numbers of HBV DNA loads [6]–[8]. These different stages of the process of CHB may be attributed to host variable immune responses.

A previous study has suggested that the failure of T cells to respond to HBV is associated with a persistent HBV replication [9]. T cell-mediated cellular immunity is involved in both viral clearance and liver injury during the HBV-infection [9], [10]. Indeed, conventional treatment of HBV-infected patients can stop or slow the progression of the disease and reduce complications, but it cannot reverse liver damage [9]. Hence, understanding the disease process and immune response is crucial for the establishment of effective therapies for CHB and reducing liver damage. Currently, the pathogenesis of virus-related chronic liver disease is not well understood, and the importance of innate and adaptive immune responses during the CHB progression is also poorly characterized.

CD4^+^ T helper cells are central regulators of immune responses, and can be classified into different subsets, according to their lineage-specific transcription factor expression, cytokine production, and subsequent immune functions [11], [12]. Notably, recent studies have demonstrated that an additional effector subset, follicular helper T (TFH) cells, is largely responsible for B cell help during an immune response, and they located in the apex of the light zone in germinal centers [13], [14]. TFH cells express chemokine receptor CXCR5, which is critical for their functions, and TFH cells also express ICOS, PD-1, and IL-21, which provide excellent markers for identification of TFH cells [14], [15], [16]. ICOS appears to be important for TFH cell development, and PD-1 is a critical regulator of the function of TFH cells and IL-21, a cytokine that is critical for the formation of germinal centers and the development of TFH cells [14], [15], [16]. Interestingly, dysregulated TFH cell function has been reported in patients with lymphoma, such as angioimmunoblastic-T-cell lymphomas (AITL), and primary cutaneous CD4^+^ small/medium-sized pleomorphic T-cell lymphoma (CSTCL) and autoimmune diseases, such as systemic lupus erythematosus (SLE) [17], [18], [19]. However, little is known on the frequency of TFH cells in CHB patients.

In this study, we explored the frequency of TFH cells in human peripheral blood from patients with CHB at IA and IT phases, and examined the potential association of the frequency of TFH cells with laboratory measures. We found a high frequency of TFH cells in CHB patients at IA stage, which was positively associated with the levels of serum AST in this population. We discussed the implications of our findings.

## Result

### High frequency of TFH cells in the peripheral blood of IA patients

**Figure 1:**
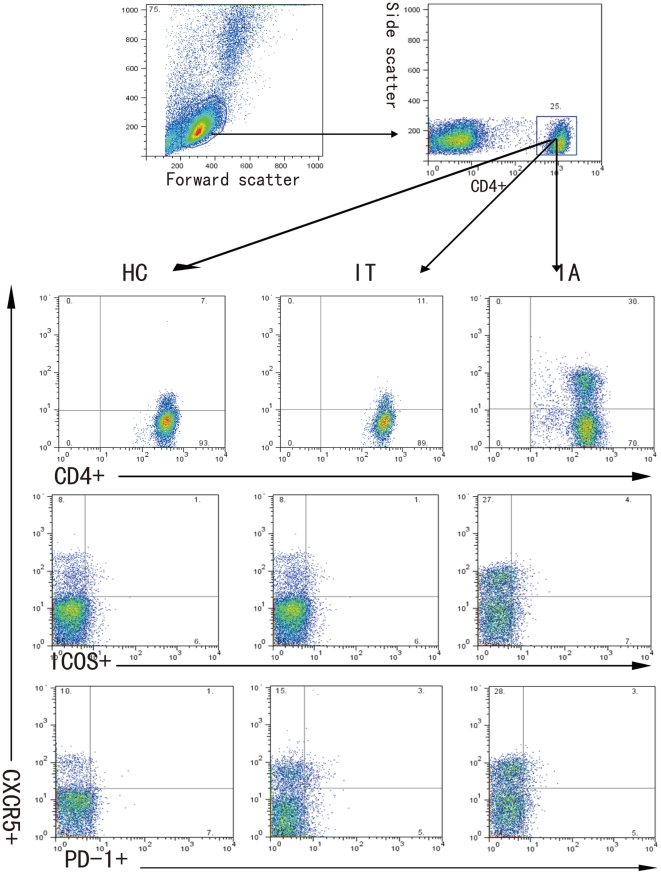
FACS analysis of TFH cells. Peripheral mononuclear cells were stained in duplicate with anti-CD4, anti-ICOS, anti-PD-1 or isotype-matched IgG, respectively. The cells were gated initially on living lymphocytes (top left) and then on CD4^+^ T cells (top right). Subsequently, the frequency of CXCR5^+^CD4^+^, ICOS^+^CXCR5^+^CD4^+^, and PD-1^+^CXCR5^+^CD4^+^ cells were analyzed by flow cytometry. At least about 50,000 events were analyzed for each sample and data are representatives of different groups of samples from at least two independent experiments.

**Figure 2:**
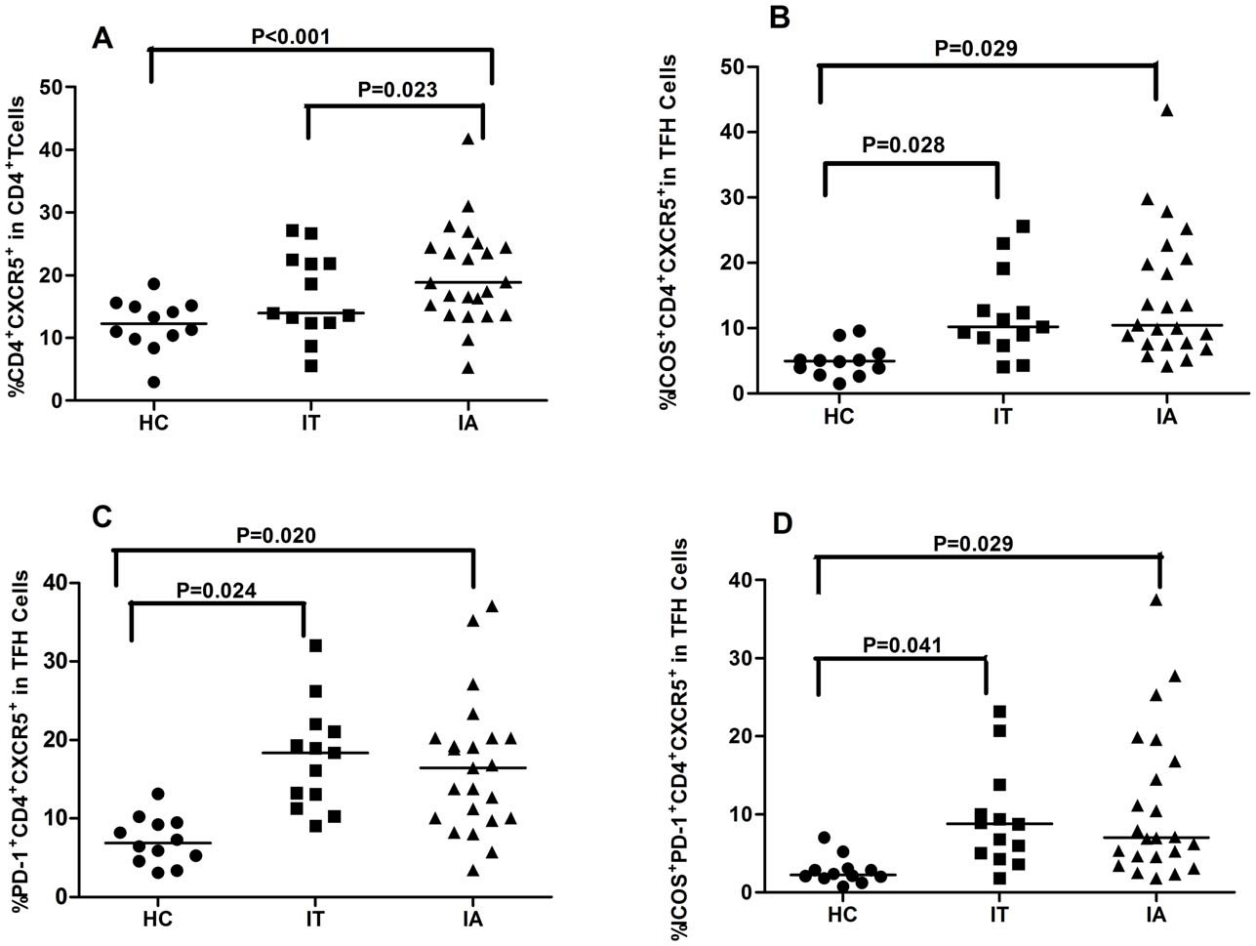
High frequency of TFH cells in the peripheral blood of IA patients. (A) The percentages of CXCR5^+^CD4^+^ in the total CD4^+^ T cells. (B) The percentages of ICOS^+^CXCR5^+^CD4^+^ in total CXCR5^+^CD4^+^ cells. (C) The percentage of PD-1^+^CXCR5^+^CD4^+^ in total CXCR5^+^CD4^+^ cells. (D) The percentage of ICOS^+^PD-1^+^CXCR5^+^CD4^+^ in total CXCR5^+^CD4^+^ cells. Data are expressed as mean % of individual samples from at least two separate experiments. The horizontal lines show the median.

**Figure 3:**
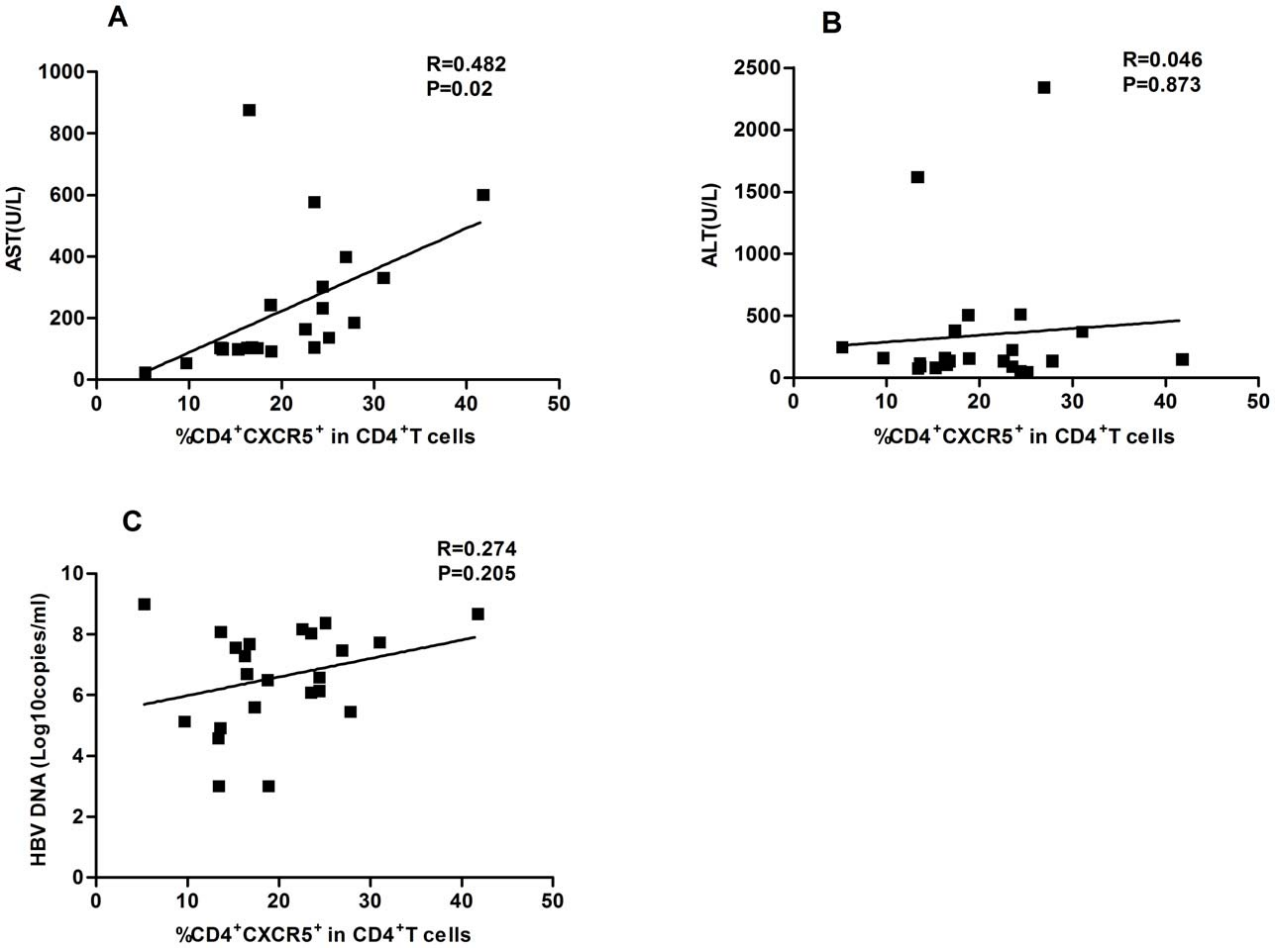
The correlation of the levels of serum AST, ALT, HBV DNA load with CXCR5^+^CD4^+^ TFH cells in drug-response IA patients. (A) AST. (B) ALT. (C) HBV DNA load. Data are expressed as mean values of individual patients (n = 27) from three separate experiments.

To determine T cell immunity, 23 IA and 13 IT patients and 12 healthy subjects were recruited. As shown in Table 1, there was no significant difference in the distribution of age and gender in this population. As expected, the levels of serum ALT and AST in IA patients were significantly higher than that of IT patients and healthy subjects, while the levels of HBV DNA loads in IA patients were significantly lower than that of IT patients. To investigate the potential role of peripheral TFH cells in HBV-infection patients, the frequency of peripheral blood CD4^+^CXCR5^+^ in CD4^+^ T cells and the percentages of ICOS^+^CD4^+^CXCR5^+^ and PD-1^+^CD4^+^CXCR5^+^ in TFH cells were analyzed by flow cytometry (Fig. 1). Interestingly, the percentages of CD4^+^CXCR5^+^ TFH cells in IA patients were significantly higher than that in IT patients (P = 0.023) and healthy individuals (P < 0.001, Fig. 2A). Furthermore, the percentages of ICOS^+^CD4^+^CXCR5^+^, PD-1^+^CD4^+^CXCR5^+^, and ICOS^+^PD-1^+^CD4^+^CXCR5^+^ TFH cells was similar between IA and IT patients, although they were significantly higher than that of healthy subjects (P< 0.05, Fig. 2B–D). There was no significant difference in the concentrations of serum IL-21 between IA and IT patients (data not shown). Further stratification indicated that there was no significant difference in the frequency of CD4^+^CXCR5^+^ TFH cells between IA patients with positive HBeAg and negative HBeAg (data not shown). More importantly, Spearman's correlation analysis revealed that the frequency of CD4^+^CXCR5^+^ TFH cells was significantly correlated with the concentrations of AST (r = 0.482, P = 0.02, Fig. 3A), but not with ALT (Fig. 3B) and the levels of HBV DNA loads (Fig. 3C) in IA patients. Apparently, the high frequency of CD4^+^CXCR5^+^ TFH cells was associated with levels of serum AST in IA patients in this chinese population.

**Table 1:** The demographic and clinical characteristics of subjects.

Parameters	Immune-active (IA)	Immune-tolerant (IT)	Healthy controls
NO.	23	13	12
Age (years)			
Mean ± SD	42.9±8.1	40.8±6.7	35.75±12.35
Median (range)	45 (30–61)	38 (30–58)	35 (23–50)
Sex N (%)			
Male	19 (83)	9 (69)	9 (75)
Female	4 (17)	4 (31)	3 (25)
HBV DNA (log10 copies/ml)			
Median (range)	4.8* (3.5–6.8)	6.9 (5.2–7.5)	NA
ALT (U/L)			
Median (range)	135* (46–2343)	30 (17–40)	15 (5–20)
AST (U/L)			
Median (range)	104.5* (24–876)	24 (21–39)	11 (9–25)

Normal values: ALT≤40 IU/L; AST≤40 IU/L; HBV DNA ≤3 log10 copies/ml;

*P<0.05 vs. IA patients.

### Treatment with Adefovir dipivoxil significantly reduces the frequency of CD4^+^CXCR5^+^ TFH cells in IA patients

**Figure 4:**
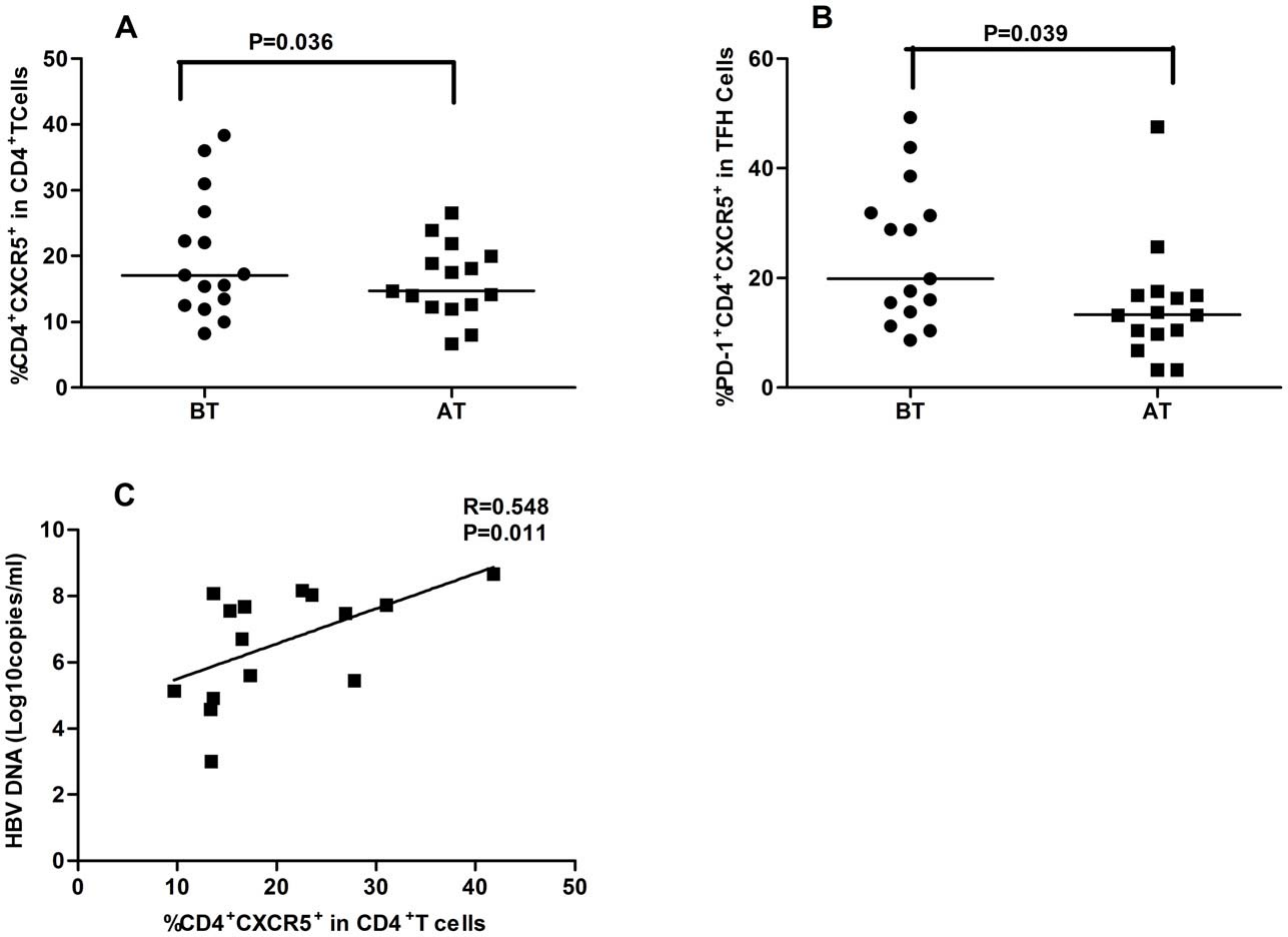
Treatment with adefovir dipivoxil modulates the frequency of TFH cells in IA patients. A total of 23 IA patients were treated with adefovir dipivoxil for 12 weeks and the frequency of TFH cells in peripheral blood was determined by flow cytometry analysis before and after treatment. (A) The percentage of CXCR5^+^CD4^+^ TFH cells in the total CD4^+^T cells; (B) The percentage of PD-1^+^CXCR5^+^CD4^+^ cells in total CXCR5^+^CD4^+^ cells. Data are expressed as mean % of individual patients who were drug responders (n = 15). The horizontal lines indicate the median values of different groups. (C) The correlation of the frequency of CXCR5^+^CD4^+^ cells with the amount of serum HBV DNA in drug-responding patients before drug treatment.

Those 23 IA patients were treated with adefovir dipivoxil for 12 weeks, and their percentages of CD4^+^CXCR5^+^ TFH cells were characterized before and after drug treatment, respectively. There were 15 IA patients responding to drug, while the other 8 patients did not (Table 2). Evidentially, following treatment with adefovir dipivoxil, all patients displayed reduced levels of serum ALT and AST, but the levels of serum HBV DNA loads only in those drug response IA patients were reduced significantly, as compared with that of before treatment. Notably, the frequency of CD4^+^CXCR5^+^ TFH cells was correlatively positively with the amounts of serum HBV DNA in those drug-responding patients before drug treatment (R^2^ = 0.3005, P = 0.011, Fig. 4C). Further analysis of TFH cells indicated that, in comparison with that before treatment, the frequency of CD4^+^CXCR5^+^ and PD-1^+^CD4^+^CXCR5^+^, but not ICOS^+^CD4^+^CXCR5^+^ and ICOS^+^PD-1^+^CD4^+^CXCR5^+^ THF cells, was also significantly reduced in drug responded IA patients (P< 0.05 for both, Fig. 4A, B). In contrast, there was no significant difference in the frequency of CD4^+^CXCR5^+^, PD-1^+^CD4^+^CXCR5^+^, ICOS^+^CD4^+^CXCR5^+^ and ICOS^+^PD-1^+^CD4^+^CXCR5^+^ THF cells in drug non-response IA patients (data not shown). Clearly, treatment with adefovir dipivoxil inhibited the replication of HBV and demolished CD4^+^CXCR5^+^ and PD-1^+^CD4^+^CXCR5^+^ THF cells in IA patients.

**Table 2:** Treatment with adefovir dipivoxil modulates the clinical profiles of IA patients.

Group	Drug responded (n = 15)		Drug non-response (n = 8)	
	Before	After	Before	After
ALT (U/L)	125 (46–2343)	42 (23–69)*	130 (112–1138)	68 (39–127)*
AST (U/L)	104.5 (24–787)	39 (23–47)*	89 (48–876)	35 (16–65)*
HBVDNA (log10copies/ml)	6.0 (5.2–7.0)	2.5 (0.3–3.1)*	5.8 (3.7–7.5)	4.9 (2.4–6.7)
HBsAg (IU/ml)	5002.04 (1243.65–55925.04)	3478.45 (890.34–17118.09)	4239.8 (224.59–22160.14)	4965.49 (654.8–18420.66)
HBsAb (mIU/ml)	0.01 (0–3.71)	0.4 (0–4.41)	0 (0–3.2)	0.55(0–3.85)
HBeAg (S/CO)	2189.6 (3.88–4094.10)*	539.62 (0.47–2345.67)*	555.855 (1.311–5573.35)	284.626 (3.24–4167.3)
HBeAb (S/CO)	16.71(0.02–46.74)*	1.26(0.01–35.67)*	32.55 (0.83–44.76)	13.23 (1.16–32.59)

Data are expressed as median (range) or real case numbers.

*P<0.05 vs. before treatment.

### Treatment with adefovir dipivoxil modulates serum cytokines and HBV-related humoral responses in drug response patients

**Figure 5:**
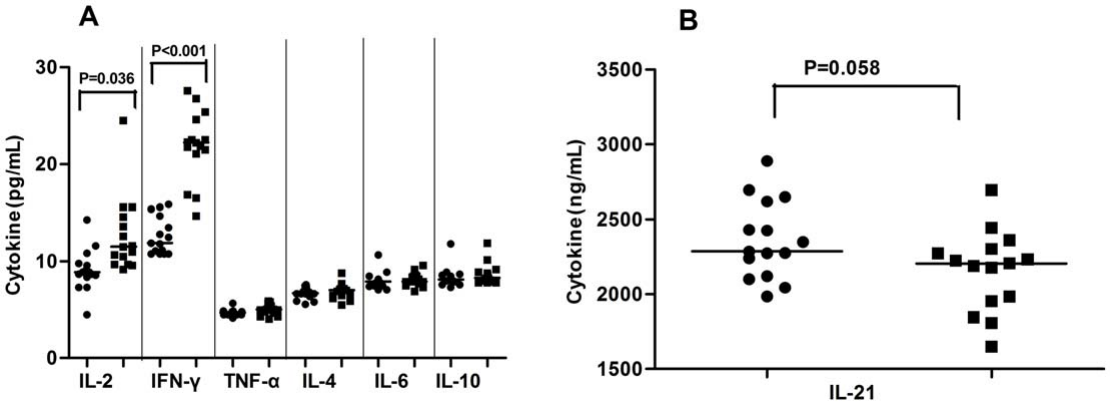
Analysis of serum cytokines in drug-response IA patients. The concentrations of serum TH1/TH2 (A), TFH (B) cytokines in drug-response IA patients before (black circle) and after (black square) adefovir dipivoxil treatment were examined by CBA. Data are expressed as mean values of individual patients (n = 15) from two separate experiments. The horizontal lines indicate the median values of different groups.

To further understand the effect of treatment with adefovir dipivoxil, we detected the concentrations of serum cytokines before and after drug treatment by cytometric bead array (CBA) and ELISA. We found that the concentrations of IL-2 and IFN-γ, but not IL-4, IL-6, IL-10, IL-21, and TNF-α, after treatment with adefovir dipivoxil were significantly higher than that before treatment in those patients (Fig. 5). However, there was no significant difference in the concentrations of serum cytokines tested in those drug non-response patients (data not shown). Furthermore, the changes in the concentrations of serum cytokines were not associated with HBV DNA loads, ALT, and AST (data not shown). Apparently, treatment with adefovir dipivoxil modulated systemic cytokine responses in patients with CHB at IA phase.

Further characterization of serum HBsAg, HBsAb, HBeAg, and HBeAb revealed that the concentrations of serum HBeAb were correlated negatively with the frequency of CD4^+^CXCR5^+^ TFH cells in patients with CHB at IA phase (r = −0.479, P = 0.013). Furthermore, treatment with adefovir dipivoxil slightly reduced the levels of serum HBsAg but increased HBsAb, but did not reach a statistically significant difference between before and after drug treatment (Table 2). In addition, treatment with adefovir dipivoxil significantly decreased the levels of serum HBeAg, but increased the levels of serum HBeAb in drug-responding patients, but not in drug non-responsding patients. Collectively, treatment with adefovir dipivoxil modulated systemic cytokine and HBV-related immune responses.

### High frequency of spelnic and liver CD4^+^CXCR5^+^ TFH Cells in HBV-transgenic mice

**Figure 6:**
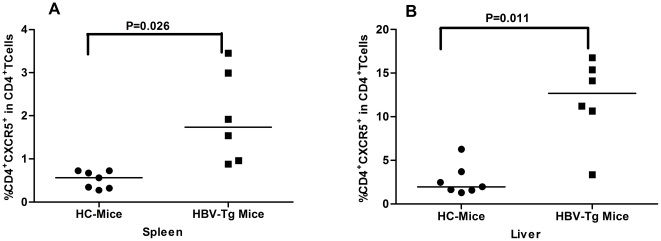
High frequency of TFH cells in the livers and spleens of HBV transgenic mice. HBV transgenic (n = 7) and wild-type (n = 6) of C57BL/6 mice at eight weeks of age were sacrificed, and their liver and splenic mononuclear cells were prepared. The frequency of splenic and liver CD4^+^CXCR5^+^ T cells was determined by flow cytometry analysis. (A) The percentage of splenic TFH cells. (B) The percentage of liver TFH cells. Data are expressed as mean % of individual mice from each group, and the horizontal lines indicate the median values of each group of mice.

HBV transgenic mice display many characteristics, similar to that in CHB patients, providing an excellent model for the evaluation of spontaneous immune response. We further characterized the frequency of splenic and liver CD4^+^CXCR5^+^ THF cells in HBV transgenic and wild-type mice. We found that the frequency of splenic and liver CD4^+^CXCR5^+^ THF cells in HBV transgenic mice was significantly higher than those in wild-type C57BL/6 mice (p < 0.05 for both, Fig. 6). Therefore, the high frequency of CD4^+^CXCR5^+^ THF cells may reflect an active status of CHB patients.

## Discussion

TFH cells are crucial regulators and have been associated with the pathogenic process of many diseases in humans [17]–[19]. The present study characterized the frequency of peripheral TFH cells in IA and IT CHB patients and HC, and revealed that the frequency of peripheral blood CD4^+^CXCR5^+^ TFH cells in IA patients was significantly higher than that of IT patients and HC. In addition, treatment with adefovir dipivoxil for 12 weeks significantly reduced the percentage of CD4^+^CXCR5^+^ TFH cells in drug-responding IA patients. Finally, the percentages of splenic and liver CD4^+^CXCR5^+^ TFH cells in HBV-transgenic mice were significantly higher than that of wild-type mice. These findings clearly indicate that TFH cells participate in the HBV-related immune responses.

The persistent HBV-infection in CHB patients is usually associated with quantitative and qualitative exhaustion of functional T cells [20]. The frequency of peripheral blood TFH cells in CHB patients varies at different stages of the process of chronic HBV-infection. Simpson and colleagues found that high frequency of peripheral blood TFH cells was detected in patients with active systemic lupus erythematosus (SLE) and Sjogren's syndrome (SS) and they also expressed ICOS, CXCR5, and PD-1 [19]. In this study, we found a higher frequency of CD4^+^CXCR5^+^ TFH cells in IA patients and increased frequency of ICOS-, PD-1-expressing CD4^+^CXCR5^+^ in CHB patients. Our data support the notion that TFH cells can circulate in peripheral blood [21]. The increased frequency of TFH cells may reflect active immune responses because TFH cells are crucial for antigen-specific B cell development and humoral responses against virus infection. Indeed, TFH cells have been thought to be resting memory TH cells [21]. However, we found that the frequency of CD4^+^CXCR5^+^ TFH cells was correlated negatively with the concentrations of serum HBeAb in CHB patients and that treatment with adefovir dipivoxil reduced the frequency of CD4^+^CXCR5^+^ TFH cells, but increased the levels of serum HBeAb, accompanied by increased levels of serum Th1 cytokines in drug-responding patients. Apparently, high frequency of TFH cells is associated with a poor immunity against HBV infection. Alternatively, the increased frequency of peripheral blood TFH cells may stem from the altered distribution of Th1 and Th2 cells due to their infiltration in the target organs [22], [23]. However, we found that the percentages of splenic and liver CD4^+^CXCR5^+^ TFH cells in HBV-transgenic mice were significantly higher than that of wild-type mice, indicating that CD4^+^CXCR5^+^ TFH cells also migrated into the target organ in this model. More importantly, we found a positive correlation between the percentages of peripheral blood CD4^+^CXCR5^+^ TFH cells and the concentrations of serum AST in IA patients. This positive correlation further suggests that TFH cells are associated with the HBV-related damages in the liver and indicates that the frequency of TFH cells may be another valuable biomarker (in addition to AST, ALT and HBV DNA loads) for distinguishing CHB patients at IA from IT phase. The high frequency of TFH cells may be a valuable biomarker for the evaluation of immune status in CHB patients at clinic. Notably, there was no significant correlation of the frequency of CD4^+^CXCR5^+^ TFH cells with the levels of serum ALT in IA patients. This may come from the population heterogeneity and small sample size in this study.

Adefovir dipivoxil is a potent antiviral reagent, and treatment with adefovir dipivoxil can effectively inhibit the replication of HBV in the majority of CHB patients. Our previous studies have shown that treatment with adefovir dipivoxil enhanced T cell immunity, which was associated with the inhibition of HBV replication in CHB patients [22], [23]. In this study, we further examined the impact of treatment with adefovir dipivoxil on systemic cytokine responses and found that treatment with adefovir dipivoxil significantly elevated the concentrations of serum IL-2 and IFN-γ, but did not affect the levels of serum IL-4, IL-6, IL-10, IL-21, and TNF-α in drug-responding patients. These results suggest that adefovir dipivoxil inhibits HBV DNA replication and promotes Th1 responses. Interestingly we found that treatment with adefovir dipivoxil for 12 weeks not only significantly decreased the concentrations of serum HBsAg, HBeAg, ALT, AST, and HBV virus loads, but also dramatically reduced the frequency of TFH cells, particularly for PD-1^+^CD4^+^CXCR5^+^ TFH cells, in the drug-responding IA patients. Treatment with adefovir dipivoxil also increased the levels of serum HBeAb in those patients. However, this treatment only slightly reduced the values of clinical measures and the frequency of TFH cells in the drug non-responding IA patients. Engagement of PD-1 by PDL1 in activated T cells usually mediates a negative signal for T cell function, and the levels of PD-1 expression are negatively associated with the activities of CD8^+^ T cells in CHB patients [20]. The decreased frequency of PD-1^+^CD4^+^CXCR5^+^ by adefovir dipivoxil treatment may be associated with increased T cell immunity in CHB patients. Alternatively, the decreased frequency of PD-1^+^CD4^+^CXCR5^+^ TFH cells may come from dramatically reduced CHB virus loads. Notably, the frequency of CD4^+^CXCR5^+^TFH cells was correlated positively with the levels of HBV DNA loads in drug-responding IA patients, but negatively with the concentrations of serum HBeAb in CHB patients. Treatment with adefovir dipivoxil reduced the frequency of CD4^+^CXCR5^+^ TFH cells, but increased the levels of serum HBeAb in drug-response IA patients. Our data are consistent with previous findings that the frequency of CD4^+^CXCR5^+^ TFH cells is associated negatively with the frequency of plasma cells [13]. The precise relationship between the frequency of peripheral blood CD4^+^CXCR5^+^ TFH cells and the reduced HBV loads, enhanced frequency of other T cells, and the increased of antibody after adefovir dipivoxil treatment remains to be further investigated.

In summary, our data indicated that there was a higher frequency of ICOS- and PD-1-expressing CD4^+^CXCR5^+^ TFH cells in CHB patients and that the frequency of peripheral blood CD4^+^CXCR5^+^ TFH cells in IA patients was significantly higher than that of IT patients. More importantly, the percentages of TFH cells were positively associated with the concentrations of serum AST in IA patients. These novel findings suggest that TFH cells participate in the HBV-related immune responses and that high frequency of TFH cells may be a valuable prognostic biomarker for the evaluation of immune statuses of CHB patients. We recognized that this study had limitations of small sample size and the lack of functional study of TFH cells in the pathogenic process of CHB and the HBV-related immunity. Therefore, further study of the function of TFH cells in the pathogenic process and HBV-related immunity with a bigger population is warranted.

## Materials and Methods

### Patients

A total of 36 patients with HBV infection were recruited in the inpatient service and another 12 healthy subjects were from the outpatient service of the First Hospital of Jilin University from Mar 2009 to Dec 2010. Individual subjects with HBV infection were confirmed positive for HBsAg and detectable HBV virions for at least 12 months [24]. Subjects with positive hepatitis C and D, HIV infection, with autoimmune hepatitis or metabolic liver disease, receiving immunosuppressive therapy, or antiviral therapy within the past 12 months before entry were excluded [24]. All of the patients denied to being drug users, or having been exposed to hepatotoxin [24]. Those HBV infected subjects were further classified into two distinct groups, according to the levels of serum HBV DNA loads and ALT. Subjects with high copies of serum HBV DNA loads and normal levels of ALT (normal range: ≤40 U/L) were considered as IT, but those with relatively low levels of serum HBV DNA loads and abnormal levels of ALT were defined as IA, as described previously [6]–[8], [25]. Their demographic and clinical characteristics are summarized in Table 1.

Those IA patients were treated orally with 10 mg of adefovir dipivoxil (Gilead Science, Forster City, USA) daily for 12 weeks. Their serum ALT, AST, HBsAg, HBsAb, HBeAg, HBeAb concentrations, and HBV DNA loads were analyzed (Table 2). Individual IA patients with at least 100-fold reduced serum HBV viral loads were defined as drug response patients, but others were defined as drug non-response patients. The study conformed for the guidelines of the Declaration of Helsinki and was approved by Human Ethics Committee of Jilin University,ChangChun,China. Written informed consent was obtained from each participant.

Peripheral blood samples were obtained from individual subjects, and the levels of serum AST and ALT were detected by Biochemistry Automatic Analyzer (Roche Diagnostics, Branchburg, USA) [22]. The levels of serum HBV DNA loads were measured by quantitative PCR assay using the luciferase quantization detection kit with a detection limit of 300 copies/mL (Roche Amplicor, Basel, Switzerland), according to the manufacturers' instruction [22]. The levels of HBV-related HBsAg, HBsAb, HBeAg, and HBeAb were determined by a chemiluminescent microparticle immunoassay (CMIA) using an Abbott I 2000 automated chemiluminescence immunoassay analyzer (Abbott Laboratories, Abbott Park, Illinois, USA). The concentrations of serum HBeAb in individual samples were determined semi-quantitatively by a competitive inhibition method, according to the manufacturers' instruction and a previous report [26]. The data are expressed as median (range) of signal OD to cut-off (S/CO). Accordingly, the higher concentrations of serum HBeAb, the lower values of S/CO.

### Mice

Both female and male C57BL/6 HBV-transgenic mice and non-transgenic C57BL/6 mice at 8 weeks of age were purchased from Vital River Laboratories (Beijing, China). This HBV-transgenic line of mice displays high levels of serum HBV replicative DNA, with the log value of the HBV DNA load [5.94, (5.45–6.40)], and all forms of HBsAg particles, which mimics the pathogenic process in human patients with CHB [27]. All mice were housed in a specific pathogen-free facility. The animal experiment was carried out in strict accordance with the recommendations in the Guide for the Care and Use of Laboratory Animals of the National Institutes of Health. The protocol was approved by the Animal Research and Protection Committee of Jilin University, Changchun, China (SYXK-2010-0008). The mice were sacrificed, and their livers and spleens were dissected out. The hepatic mononuclear cells (HMNCs) were prepared by meshing and Percoll gradient centrifuging. Briefly, the liver tissue samples were meshed through a 200-gauge stainless steel filter, and after being washed, the liver cells were centrifuged and re-suspended in 40% Percoll (Pharmacia, Uppsala, Switzerland). Subsequently, the cell suspension was overlaid gently on the top of 70% Percoll and centrifuged at 2,400 rpm for 30 min at room temperature [28]. HMNCs were obtained from the interphase and washed twice with PBS. The remaining erythrocytes were removed using lysis solution (Beckton Dickinson, San Jose, USA).

### Flow cytometry

Peripheral blood mononuclear cells (PBMCs) were isolated by density-gradient centrifugation using Ficoll-Paque Plus (Amersham Biosciences, Little Chalfont, UK). Human PBMCs at 10^6^/tube were stained in duplicate with PerCP-anti-CXCR5 (Biolegend, San Diego, USA) and APC-anti-CD4, PE-anti-CD278, FITC-anti-CD279, or isotype-matched control IgG (Beckton Dickinson, San Jose, USA) at room temperature for 30 minutes, respectively. After being washed with PBS, the cells were subjected to flow cytometry analysis using a FACSCalibur (Beckton Dickinson) and FlowJo software (v5.7.2) [22]. The cells were gated on the forward scatter of living cells and then centered on CD4^+^ T cells. Subsequently, the CD4^+^CXCR5^+^, ICOS^+^CD4^+^CXCR5^+^, PD-1^+^CD4^+^CXCR5^+^, and ICOS^+^PD-1^+^CD4^+^CXCR5^+^ TFH cells were determined by flow cytometric analysis, and at least 50,000 events per sample were analyzed.

Additional flow cytometry analysis was performed for mouse splenic and hepatic mononuclear cells. Briefly, splenic or hepatic mononuclear cells at 10^7^/tube were stained in duplicate with FITC-anti-CD4 (eBioscience, San Diego, USA) and PE-anti-CXCR5 (BD Pharmingen, San Diego, USA), and the frequency of CD4^+^CXCR5^+^ TFH cells was determined by flow cytometry analysis.

### Enzyme-linked ImmunoSorbent assay (ELISA)

The concentrations of serum IL-21 in individual patients and HC were determined by ELISA using human IL-21 ELISA Kit, according to the manufacturers' instruction (Roche Diagnostics, Lewes, UK). Individual sera at 1∶4 dilutions were subjected to ELISA analysis, and the concentrations of serum IL-21 in individual samples were calculated, according to the standard curve.

### Cytometric bead array (CBA) analysis of serum cytokines

The concentrations of serum cytokines were determined by CBA [29], [30], according to the manufacture's protocol (CBATM, BD Biosciences, San Joes, USA). The concentrations of cytokines in individual samples were quantified in duplicate using the CBA kit on a FACSCalibur cytometry (BD Biosciences) equipped with CellQuestPro and CBA software (Becton Dickinson).

### Statistical analysis

Data are expressed as median and range unless specified. The difference between two groups was analyzed by Wilcoxon rank sum test and Chi-square test using the SAS version 8.0 software. The relationship between two variables was evaluated using the Spearman rank correlation test. A two-side P value < 0.05 was considered statistically significant.

## References

[1] Wang FS, Xing LH, Liu MX, Zhu CL, Liu HG (2001). Dysfunction of peripheral blood dendritic cells from patients with chronic hepatitis B virus infection.. World J Gastroenterol.

[2] Bertoletti A, Gehring AJ (2006). The immune response during hepatitis B virus infection.. J Gen Virol.

[3] Pol S (2006). Natural history of hepatitis B infection.. Presse Med.

[4] Baumert TF, Thimme R, von Weizsäcker F (2007). Pathogenesis of hepatitis B virus infection.. World J Gastroenterol.

[5] Lai CL, Yuen MF (2007). The natural history of chronic hepatitis B.. J Viral Hepat.

[6] Rehermann B, Nascimbeni M (2005). Immunology of hepatitis B virus and hepatitis C virus infection.. Nat Rev Immunol.

[7] Pan CQ, Zhang JX (2005). Natural history and clinical consequences of hepatitis B virus infection.. Int J Med Sci.

[8] Ganem D, Prince AM (2004). Hepatitis B virus infection-natural history and clinical consequences.. N Engl J Med.

[9] Stoop JN, van der Molen RG, Baan CC, van der Laan LJ, Kuipers EJ (2005). Regulatory T cells contribute to the impaired immune response in patients with chronic hepatitis B virus infection.. Hepatol.

[10] Franzese O, Kennedy PT, Gehring AJ, Gotto J, Williams R (2005). Modulation of the CD8+-T-cell response by CD4+CD25+ regulatory T cells in patients with hepatitis B virus infection.. J Virol.

[11] Pesu M (2010). T-helper cells–bandleaders of immune response.. Duodecim.

[12] Huang X, Reynolds AD, Mosley RL, Gendelman HE (2009). CD 4+ T cells in the pathobiology of neurodegenerative disorders.. J Neuroimmunol.

[13] Pelletier N, McHeyzer-Williams LJ, Wong KA, Urich E, Fazilleau N (2010). Plasma cells negatively regulate the follicular helper T cell program.. Nat Immunol.

[14] Laurent C, Fazilleau N, Brousset P (2010). A novel subset of T-helper cells: follicular T-helper cells and their markers.. Haematologica.

[15] Linterman MA, Vinuesa CG (2010). Signals that influence T follicular helper cell differentiation and function.. Semin Immunopathol.

[16] Zhang CM, Zhang B, Zhang Y, Xu ZW, Yang K (2009). Identification of human follicular helper T cells in peripheral blood by flow cytometry.. Chin J Cell Mol Immunol.

[17] Rodríguez-Pinilla SM, Atienza L, Murillo C, Perez- Rodriguez A, Montes-Moreno S (2008). Peripheral T-cell Lymphoma With Follicular T-cell Markers.. Am J Surg Pathol.

[18] Rodríguez Pinilla SM, Roncador G, Rodríguez-Peralto JL, Mollejo M, García JF (2009). Primary Cutaneous CD4+ Small/Medium-sized Pleomorphic T-cell Lymphoma Expresses Follicular T-cell. Markers.. Am J Surg Pathol.

[19] Simpson N, Gatenby PA, Wilson A, Malik S, Fulcher DA (2010). Expansion of circulating T cells resembling follicular helper T cells is a fixed phenotype that identifies a subset of severe systemic lupus erythematosus.. Arthritis Rheum.

[20] Ye P, Weng ZH, Zhang SL, Zhang JA, Zhao L (2008). Programmed death-1 expression is associated with the disease status in hepatitis B virus infection.. World J Gastroenterol.

[21] Sallusto F, Geginat J, Lanzavecchia A (2004). Central memory and effector memory T cell subsets: function, generation, and maintenance.. Annu Rev Immunol.

[22] Jiang YF, Ma ZH, Xin GJ, Yan HQ, Li WY (2010). Th1 and Th2 immune response in chronic hepatitis B patients during a long-term treatment with adefovir dipivoxil.. Mediators Inflamm.

[23] Jiang YF, Li W, Yu L, Liu JJ, Xin GJ (2010). Enhancing the antihepatitis B virus immune response by adefovir dipivoxil and entecavir therapies.. Cell Mol Immunol.

[24] Yim HJ, Lok AS (2006). Natural history of chronic hepatitis B virus infection: what we knew in 1981 and what we know in 2005.. Hepatology.

[25] Zhang Z, Zhang S, Zou Z, Shi J, Zhao J (2011). Hypercytolytic activity of hepatic natural killer cells correlates with liver injury in chronic hepatitis B patients.. Hepatol.

[26] Li A, Yuan Q, Huang Z, Fan J, Guo R (2010). Novel double-antigen sandwich immunoassay for human hepatitis B core antibody.. Clin Vaccine Immunol.

[27] Bandi P, Garcia ML, Booth CJ, Chisari FV, Robek MD (2010). Bortezomib inhibits hepatitis B virus replication in transgenic mice.. Antimicrob Agents Chemother.

[28] Chen Y, Wei H, Sun R, Dong Z, Zhang J (2007). Increased susceptibility to liver injury in hepatitis B virus transgenic mice involves NKG2D-ligand interaction and natural killer cells.. Hepatology.

[29] Morgan E, Varro R, Sepulveda H (2004). Cytometric bead array: a multiplexed assay platform with applications in various areas of biology.. Clin Immunol.

[30] Tarnok A, Hambsch J, Chen R, Varro R (2003). Cytometric bead array to measure six cytokines in twenty-five microliters of serum.. Clin Chem.

