# Correction: High Frequency of CD4^+^CXCR5^+^ TFH Cells in Patients with Immune-Active Chronic Hepatitis B

**DOI:** 10.1371/annotation/3d53eb95-fbf2-4295-b08b-8f7347f5ad11

**Published:** 2011-08-23

**Authors:** Junyan Feng, Lu Lu, Cong Hua, Ling Qin, Pingwei Zhao, Juan Wang, Ye Wang, Wanyu Li, Xiaodong Shi, Yanfang Jiang

The affiliations for the 1st, 8th, 9th, and 10th authors were not indicated. The complete author list and affiliations are:

Junyan Feng(1,2), Lu Lu(1), Cong Hua(1), Ling Qin(1), Pingwei Zhao(1), Juan Wang(1), Ye Wang(1), Wanyu Li(2), Xiaodong Shi(2), Yanfang Jiang(1,2)

(1) Department of Central Laboratory, the Second Part of First Hospital, Jilin University, Changchun, China

(2) Department of Hepatology, First Hospital, Jilin University, Changchun, China

