# Correction: High Frequency of CD4^+^CXCR5^+^ TFH Cells in Patients with Immune-Active Chronic Hepatitis B

**DOI:** 10.1371/annotation/e020f08b-d29e-4e50-a0bd-4491899b0cf7

**Published:** 2011-08-23

**Authors:** Junyan Feng, Lu Lu, Cong Hua, Ling Qin, Pingwei Zhao, Juan Wang, Ye Wang, Wanyu Li, Xiaodong Shi, Yanfang Jiang

The authors wish to add the following grant support to the funding information: "National Natural Science Foundation of China (No. 30771912)". 

